# Elevated Creatinine Clearance in Lupus Nephritis patients with Normal Creatinine

**DOI:** 10.7150/ijms.51117

**Published:** 2021-01-29

**Authors:** Sheng Wang, Fang Wang, Xiao Wang, Yuxian Zhang, Lijun Song

**Affiliations:** Department of Rheumatology, Qilu Hospital of Shandong University, Ji'nan 250012, Shandong, China.

**Keywords:** lupus nephritis, creatinine, creatinine clearance, protein urine, urine protein/creatinine ratio

## Abstract

**Objectives:** The present study aimed to observe the differences in creatinine clearance (Ccr) in systemic lupus erythematosus (SLE) patients with normal serum creatinine at different levels of urinary protein.

**Method:** The present cross-sectional study included 177 SLE patients with normal serum creatinine from Qilu Hospital of Shandong University between January 2010 and April 2020. The following data were collected: blood urea nitrogen (BUN), serum creatinine (Cr), serum total protein, serum albumin, immunoglobulin (Ig) G, IgA, IgM, complement 3, complement 4, anti-ds-DNA antibody, routine urine test, urine protein/creatinine ratio (UPCR) (g/g), and the SLE disease activity index. The estimated Ccr was calculated according to the Cockcroft formula.

**Results:** 123 patients were with positive urinary protein (Lupus Nephritis, LN group) and 54 patients were with negative urinary protein (Non-LN group). Compared with the Non-LN group, the LN group had higher BUN (5.76±3.22 *vs*. 4.78±1.58, P=0.007) and Cr (62.36±19.53 *vs*. 54.83±11.09, P=0.001). There was a strong correlation between the UPCR and the semi-quantitative determination of urine protein in LN patients (r=0.9583, P=0.0417). The serum creatinine levels were significantly higher in patients with urine protein 3+ (72.97±25.16) or massive proteinuria (62.32±19.66) than the other groups. Patients with urinary protein ± exhibited a significantly elevated Ccr when compared to patients with urinary protein 3+ (130.6±44.15 *vs.* 110.5±33.50, P=0.02), and patients with UPCR<0.15 g/g had higher Ccr than other groups and showed significantly increased Ccr compared with patients with UPCR≥0.15 g/g (132.44±21.02 *vs.* 115.14±35.89, P=0.007).

**Conclusions:** Early renal function impairment may be present in LN patients. The kidneys of LN patients with urinary protein ± or UPCR<0.15 g/g are in a state of hyperfunction.

## Introduction

Systemic lupus erythematosus (SLE) is an autoimmune disease that involves multiple body systems and multiple autoantibodies. Renal involvement is one of the most common and serious manifestations of SLE 1. Despite the improvement in its treatment, there is still an increase in mortality for people with SLE, when compared to matched controls 2, 3.

It has long been hoped that certain clinical manifestations could predict the pathological damage to the kidney without the need for renal biopsy, thereby enabling the early diagnosis and selection of appropriate treatment options. However, according to previous research, no consistent clinical-pathological relationship can predict the patterns or severity of histological findings based on clinical renal manifestations 4, 5.

The assessment of the glomerular filtration rate (GFR) is essential in clinical practice 6. In recent years, several new equations have been developed to estimate the GFR, such as the Modification of Diet in Renal Disease (MDRD) equation 7, the Schwartz equation 8, and the Chronic Kidney Disease Epidemiology Collaboration (CKD-EPI) equation 9. Although the National Kidney Foundation (NKF) suggests the use of these equations to estimate GFR, rheumatologists continue to use creatinine clearance (Ccr) 10. In fact, the Cockcroft-Gault (CG) equation 11 is still widely adopted for the estimation of creatinine clearance in rheumatic clinical practice due to its superior convenience.

The application value of Ccr in lupus nephritis (LN) patients with elevated serum creatinine and the accuracy of various formulas have been widely verified 10, 12, 13, but the value of the Ccr in LN patients with normal serum creatinine has been overlooked. As we know, diabetic nephropathy patients in the early stages of the disease will show an increased GFR 14. Whether LN patients with normal serum creatinine exhibit a similar change in the early stages of the disease as those with diabetic nephropathy remains unclear. In the present study, the investigators aimed to clarify the difference in Ccr based on various urinary protein levels, and the relationship between Ccr and urinary protein level, as well as other clinical and laboratory tests in SLE patients with normal serum creatinine.

## Methods

### Study Population

The present study is a cross-sectional study that included 177 SLE patients with normal serum creatinine from Qilu Hospital of Shandong University between January 2010 and April 2020. SLE was diagnosed according to the 1997 American College of Rheumatology (ACR) criteria 15. SLE patients with other autoimmune diseases, active infections, malignancies, and pregnant women were not included in the study. The present study was approved by the Medical Ethics Committees of Qilu Hospital of Shandong University and had been performed following the principles of the Declaration of Helsinki.

### Data Collection

The following data were collected: age, gender, blood urea nitrogen (BUN), serum creatinine (Cr), serum total protein (TP), serum albumin (Alb), immunoglobulin (Ig) G, IgA, IgM, complement 3 (C_3_), complement 4 (C_4_), and anti-ds-DNA antibody (ds-DNA). In addition, a routine blood test was performed, which included white blood cells (WBC), red blood cells (RBC), hemoglobin (HGB) and platelets (PLT). Based on the results of the routine urinary test, the urine protein was assigned one of six levels: -, ±, 1+, 2+, 3+ and 4+. Accordingly, SLE patients were defined as Non-LN (-) and LN (±, 1+, 2+, 3+ and 4+), respectively. The urine protein/creatinine ratio (UPCR) (g/g) was also collected. For the LN group, we classified all participants as having normal range proteinuria (UPCR<0.15 g/g), mild proteinuria (0.15≤UPCR<0.5 g/g), moderate proteinuria (0.5≤UPCR<3.5 g/g), and massive proteinuria (UPCR≥3.5 g/g) based on UPCR results. SLE disease activity was evaluated with SLEDAI-2K 16. The estimated Ccr was calculated according to the following formula: Ccr = (140-age [years]) × BW (kg)/72 × Pcr × 0.011 × correctional factor × (1.73/TBSA) Females: correctional factor (0.85) 11.

### Statistical Analysis

SPSS 20.0 for Windows (SPSS Inc., Chicago, Illinois, USA) was used for all the analyses. All data were analyzed using a homogeneity test and presented as mean ± standard deviation (SD) or median (range) unless otherwise stated. Independent-samples t-test or Mann-Whitney U test was used for comparing the variables between the LN group and Non-LN group. The statistical analysis among multiple groups was performed with one-way ANOVA and post-hoc multiple comparisons by Bonferroni. The correlations between variables were assessed by Pearson's or Spearman's rank correlation coefficient. The nominal *P*-value used to determine statistical significance was *P*<0.05.

## Results

### Demographic and laboratory characteristics in LN and non-LN patients

54 patients were in the Non-LN group and 123 patients were in the LN group. The demographic characteristics and laboratory measurements are shown in Table 1. Compared with Non-LN patients, LN patients had higher BUN (P=0.007), Cr (P=0.001) and anti-dsDNA antibody (P<0.001), and lower TP (P<0.001), Alb (P<0.001) and C_4_ (P=0.023). There were no significant differences in complete blood count, including WBC, RBC, HGB and PLT, between Non-LN and LN patients. There was also no significant difference in the disease activity index (SLEDAI) between the two groups. Because most patients with urinary protein 4+ presented with elevated serum creatinine, they were not included in this study.

### The comparisons of urine protein/creatinine ratio (UPCR) based on the semi-quantitative determination of urinary protein

**Figure 1:**
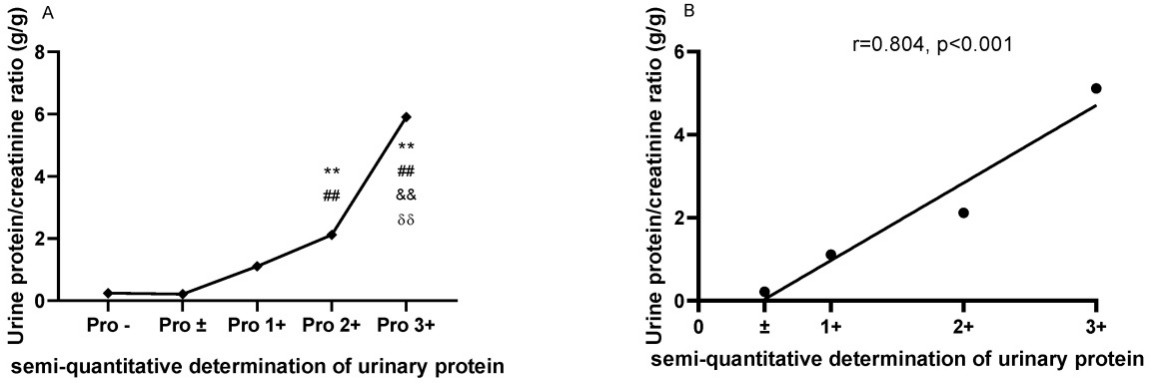
** The comparisons and correlation between the urine protein/creatinine ratio (UPCR) and the semi-quantitative determination of urinary protein. A.** The comparisons of the urine protein/creatinine ratio (UPCR) based on the semi-quantitative determination of urinary protein. **B.** The correlation between the urine protein/creatinine ratio (UPCR) and the semi-quantitative determination of urinary protein. The plots indicate the means of the urine protein/creatinine ratio. The statistical significance for the differences among multiple groups was determined using ANOVA. **P<0.01 compared to Pro -; ##P<0.01 compared to Pro ±; ^&&^P<0.01 compared to Pro 1+; ^δδ^P<0.01 compared to Pro 2+.

As shown in Figure 1A, the UPCRs were higher in patients with urinary protein 2+ (2.114±1.666) and 3+ (5.904±4.642) than in patients with urinary protein - (0.247±0.348) and ± (0.214±0.201). Meanwhile, the UPCR was higher in patients with urinary protein 3+ (5.112±2.444) than in patients with urinary protein 1+ (1.108±1.538) and 2+ (2.114±1.666). There was a strong correlation between the UPCR and semi-quantitative determination of urine protein in LN patients (r=0.9583, P=0.0417; Figure 1B).

### Comparison of serum creatinine based on the semi-quantitative determination of urinary protein and UPCR

**Figure 2:**
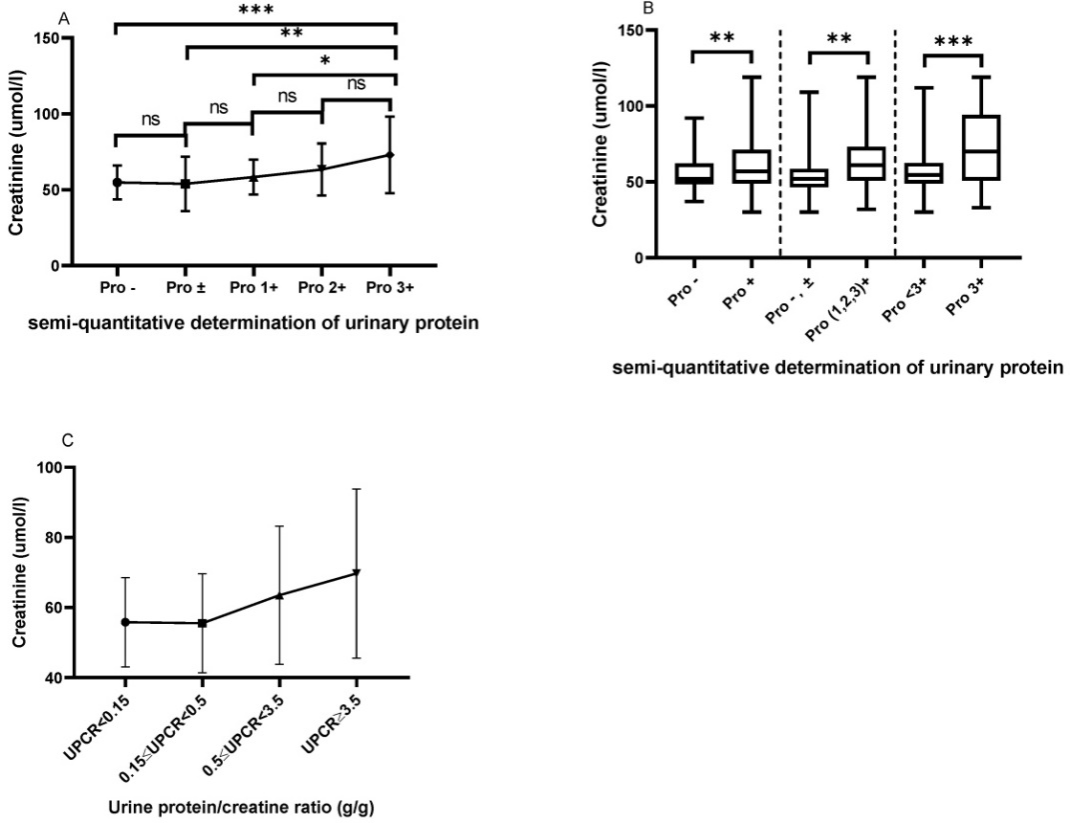
** The comparison of serum creatinine based on the semi-quantitative determination of urinary protein. A.** The Cr levels from Pro- to Pro 3+. **B.** The comparison of Cr based on the different urinary protein groups. **C.** The Cr levels based on UPCR in the LN group. The statistical significance for the differences among multiple groups was determined using ANOVA. *P<0.05, **P<0.01, ***P<0.001.

As shown in Figure 2A, patients with urinary protein 3+ had higher serum creatinine levels than those with urinary protein - (72.97±25.16 *vs*. 54.83±11.09, P=0.0001), ± (72.97±25.16 *vs*. 53.96±17.91, P=0.0011), and 1+ (72.97±25.16 *vs*. 58.33±11.43, P=0.0244). Figure 2B shows that patients with positive urinary protein (1+, 2+ and 3+) had a higher serum creatinine level than those with urinary protein - and ± (64.72±19.40 *vs*. 54.54±13.63, P=0.0036). Meanwhile, the serum creatinine levels in patients with urinary protein 3+ were significantly higher than in those with urinary protein <3+ (72.97±25.16 *vs*. 57.32±14.37, P=0.0002). We classified patients according to the UPCR, and with the increase of proteinuria, the serum creatinine of the patients showed a trend of gradual increase and the difference between the different groups was statistically significant (P=0.022). Patients with massive proteinuria showed significantly increased serum creatinine levels compared with other patients (Figure 2C).

### Comparison of creatinine clearance (Ccr) based on the semi-quantitative determination of urinary protein and UPCR

**Figure 3:**
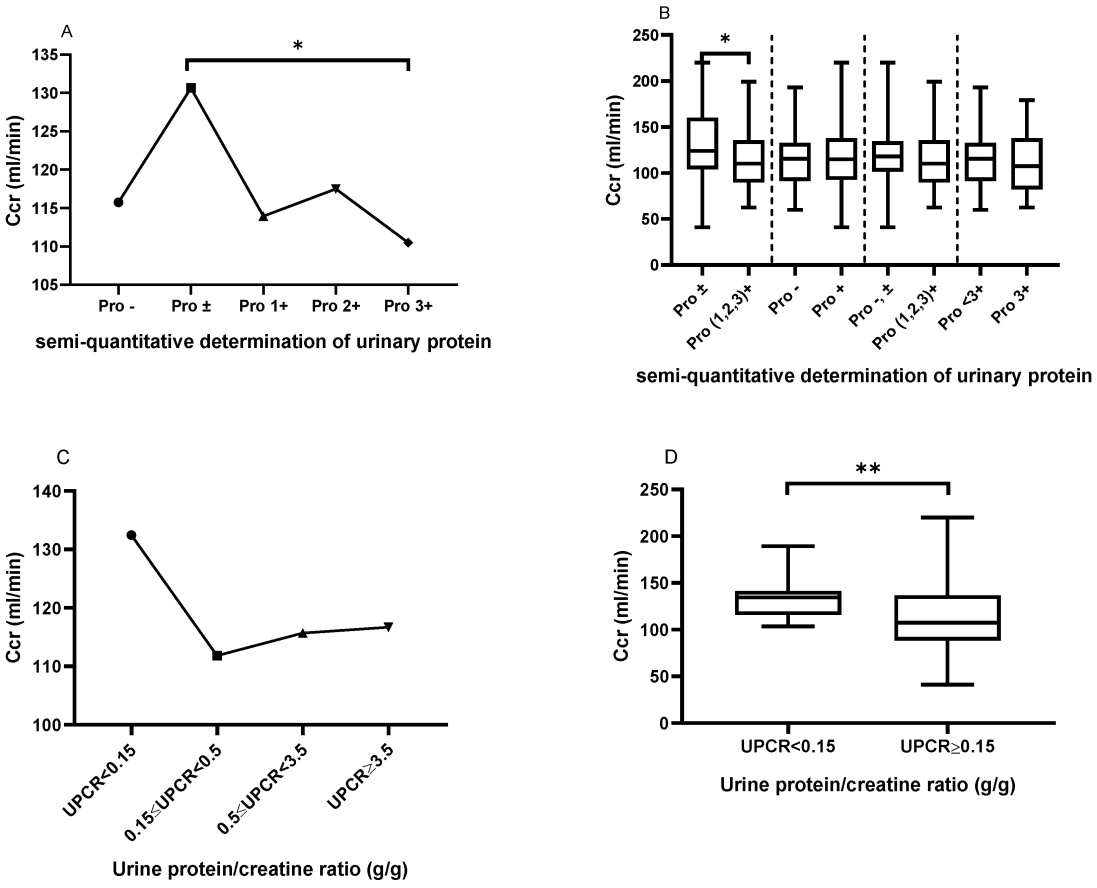
** The comparison of creatinine clearance (Ccr) based on the semi-quantitative determination of urinary protein and UPCR. A.** The Ccr from Pro- to Pro 3+. The plots indicate the means of the Ccr. **B.** The comparison of Ccr based on the different urinary protein groups. **C.** The Ccr trend based on UPCR in the LN group. **D.** The comparison of Ccr between UPCR<0.15 g/g and UPCR≥0.15g/g in the LN group. The statistical significance for the differences among multiple groups was determined using ANOVA and the differences between two groups were determined using independent-samples t-test or Mann-Whitney U test. *P<0.05, **P<0.01.

As shown in Figure 3A, patients with urinary protein ± had a significantly elevated Ccr, when compared to those with urinary protein 3+ (130.6±44.15 *vs.* 110.5±33.50, P=0.02). However, there was no significant difference in creatinine clearance among the other urinary protein groups. Similarly, as shown in Figure 3B, patients with urinary protein ± had a significantly elevated Ccr, when compared to patients with urinary protein (1+, 2+ and 3+) (130.6±44.15 *vs.* 114.0±30.35, P=0.0256). However, there were no statistically significant differences in creatinine clearance between urine protein - and + (115.7±29.32 *vs.* 117.7±34.36, P=0.7210) between urine protein (- and ±) and urine protein (1+, 2+ and 3+) (120.7±35.40 vs. 114.0±30.35, P=0.1769), or between urine protein <3+ and 3+ (118.5±32.63 vs. 110.5±33.50, P=0.2196). Similarly, we classified patients in the LN group according to UPCR, and patients with UPCR<0.15 g/g showed higher Ccr compared with patients with mild proteinuria (0.15≤UPCR<0.5 g/g) (132.44±21.02 *vs.* 111.82±34.90, P=0.298), moderate proteinuria (0.5≤UPCR<3.5 g/g) (132.44±21.02 *vs.* 115.69±37.80, P=0.382), and massive proteinuria (UPCR≥3.5 g/g) (132.44±21.02 *vs.* 116.67±34.59, P=0.626) (Figure 3C). Patients with UPCR<0.15 g/g showed significantly increased UPCR≥0.15 g/g compared to those with massive proteinuria (UPCR≥3.5 g/g) (132.44±21.02 *vs.* 115.14±35.89, P=0.007) (Figure 3D).

### The correlation between Ccr and the urine protein/creatinine ratio (UPCR), disease activity indexes and blood routine test in peripheral blood

**Figure 4:**
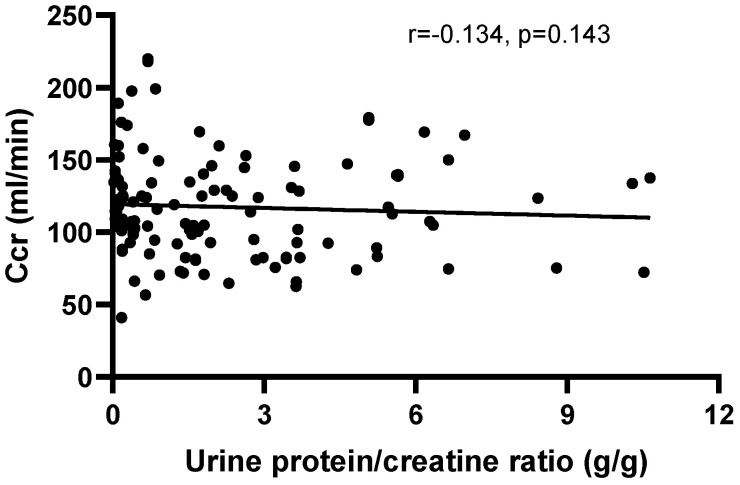
** The correlation between creatinine clearance (Ccr) and the urine protein/creatinine ratio (UPCR) in patients with positive urinary protein from Pro ± to Pro 4+.** The correlation was analyzed by Spearman's rank correlation test. P<0.05 was considered statistically significant.

There was no correlation between Ccr and the UPCR (r=-0.1383, P=0.1272) in patients with positive urinary protein from Pro ± to Pro 3+ (Figure 4). Furthermore, there was no correlation between creatinine clearance and the other disease activity indexes, including IgG (r=0.0179, P=0.8235), IgM (r=-0.0232, P=0.7727), C3 (r=0.0974, P=0.2050), C4 (r=0.0205, P=0.7897) and anti-ds-DNA antibody (r=-0.0741, P=0.3472), except for IgA (r=-0.288, P=0.002). Moreover, no correlations were observed between Ccr and HGB (r=0.0143, P=0.8494), RBC (r=0.1005, P=0.1805), PLT (r=0.1171, P=0.1184) and WBC (r=-0.0912, P=0.2246).

### The correlation between UPCR and RBC and HGB in peripheral blood

**Figure 5:**
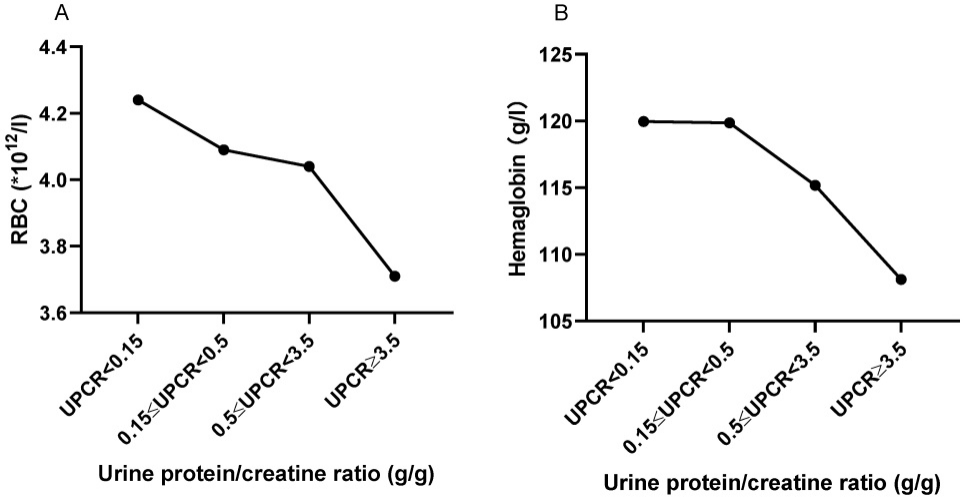
** The trend of RBC and HGB based on UPCR. A.** The RBC trend based on UPCR. **B.** The hemoglobin trend based on UPCR. The statistical significance for the differences among multiple groups was determined using ANOVA.

As shown in Figure 5, with the increase of urine protein, RBC and HGB in peripheral blood showed a trend of gradual decrease. Peripheral RBC of patients with massive proteinuria (UPCR≥3.5 g/g) was significantly decreased compared with patients with normal range (UPCR<0.15 g/g) (3.706±0.710, *vs.* 4.238±0.762, P=0.054). HGB also showed a similar trend to RBC. Also, UPCR was significantly negatively correlated with RBC (r=-0.218, P=0.016) and HGB (r=-0. 180, P=0.048).

## Discussion

Lupus nephritis (LN) is one of the most common and serious manifestations of SLE 17. Although new biomarkers such as monocyte chemoattractant protein-1 18 have been used to assess LN disease activity, conventional assays such as serum creatinine, proteinuria, GFR, urine sediments, anti-dsDNA antibody, and the complement levels, despite their inadequate sensitivity and specificity for monitoring the disease activity and early relapse in LN 17, 19, are still the most commonly used clinical indicators of LN. In the present study, the investigators attempted to elucidate the role of urinary protein, the UPCR and Ccr, as indicators of disease activity or severity in LN patients with normal serum creatinine.

The proteinuria in patients with LN plays an important role in the diagnosis, disease activity monitoring, and prognosis of LN 20, 21. The quantification of the protein content in the 24-hour urine collection sample (24hP) has been considered as the “gold standard”. However, this test is very inconvenient for most patients and is sometimes incorrectly performed 22. Recently, a strong correlation between the UPCR and 24hP in LN 13, 14 was observed in multiple studies 23, 24, and the UPCR was recommended by the American College of Rheumatology (ACR) 25 and the European League Against Rheumatism 26 for LN. In the present study, it was found that the UPCR closely correlated with the semi-quantitative determination of urine protein, which is consistent with previous studies 27, 28. Therefore, in the clinical practice of rheumatology and nephrology, the diagnosis and disease activity monitoring of LN can be determined by combining the semi-quantitative urine protein test with the UPCR for LN patients who are not suitable for 24hP.

Although serum creatinine cannot provide an adequate estimate of the glomerular filtration rate (GFR), serum creatinine remains by far the most widely used index for renal function in clinical practice and clinical trials 29, 30. The present study revealed that the serum creatinine was significantly higher in LN patients than in Non-LN patients, as well as in patients with urinary protein 3+ than in those with urinary Pro-, Pro ± and Pro 1+. Meanwhile, the serum creatinine levels were significantly higher in patients with urinary protein (1, 2, 3) + than in those with urinary protein negative and Pro ±, and higher in patients with urinary protein 3+ than in those with urinary protein <3+. These results suggest that although serum creatinine remains in the normal range in patients with urinary protein 3+, signs of renal impairment have begun to appear. Therefore, for LN patients with large amounts of proteinuria, it is necessary not only to closely monitor the experimental indicators of renal injury but also to provide active treatment to prevent irreversible renal damage.

Ccr is a rapid and cost-effective method for the measurement of renal function 31. Ccr approximates the calculation and overestimates the GFR by approximately 10%-20% 6. Although creatinine clearance is not an accurate enough measure of GFR in clinical practice, it remains as the preferred clinical indicator of renal function for rheumatologists and nephrologists in LN outpatients due to its computability. Previous studies have focused on the value of Ccr in patients with abnormal serum creatinine 10, but the significance of Ccr in patients with normal serum creatinine, especially lupus nephritis, has not been reported. A study from Spain found that patients who developed contrast nephropathy had a lower creatinine clearance rate at admission even though their serum creatinine was in the normal range 32. Another study reported that creatinine clearance increased in polytrauma patients with normal serum creatinine 33. In the present study, patients with urinary protein ± and UPCR<0.15 g/g exhibited a significant Ccr increase when compared to the other urinary protein groups, especially the urinary Pro 3+ group, and this is similar to the renal presentation of diabetic nephropathy 14. Alterations in renal function and structure were found even at the onset of diabetes mellitus, and in stages 2 and 3 of diabetic nephropathy, the glomerular filtration rate is elevated 14. Therefore, the investigators inferred that the early stages of lupus nephritis would also present with an increase in GFR, and the present findings support this deduction.

Complement system 34, anti-dsDNA antibody 35, 36, and immunoglobulins 37 are involved in the pathogenesis of SLE. Among these, C_3_, C_4_ and the anti-dsDNA antibody are closely correlated to disease activity, which was a risk factor for organ damage and was significantly positively correlated with organ damage 38. In the present study, there was no correlation between Ccr and the other disease activity indexes, including IgG, IgM, C3, C4 and the anti-ds-DNA antibody, except for IgA. Therefore, Ccr may not be a potential indicator of LN disease activity. Furthermore, the involvement of the hematologic system is a common clinical manifestation of SLE. The presence of hemolytic anemia in lupus patients is associated with a higher frequency of proteinuria and urinary cellular casts 39, and thrombocytopenia is highly comorbid with lupus nephritis 40. In the present study, we found that RBC and HGB showed a gradual downward trend with the increase of urinary protein. This suggests that when the kidney of SLE patients is involved, it is often accompanied by the involvement of the hematologic system, which requires active therapeutic intervention.

## Conclusion

In conclusion, although serum creatinine levels in SLE patients remain in the normal range, patients with urine protein have shown increased serum creatinine levels compared with patients with negative urine protein, suggesting that these patients have already presented early renal injury. At the same time, the patients with urine protein semi-quantitative ± or UPCR<0.15 had higher Ccr than other patients, suggesting that the kidneys of these patients were in a state of hyperfunction. A high Ccr does not mean that the patient is in a very good kidney condition, but rather indicates that the patient is in an early stage of the disease, and early active intervention may help prevent permanent kidney damage.

## Limitations

The present study is a single-center cross-sectional study. In the future, there is a need to expand the sample size and increase the number of research centers to further verify the conclusions of the present study. Since most patients with urinary protein 4+ presented with elevated serum creatinine, they were not included in this study and will need to be further analyzed in the future.

## Figures and Tables

**Table 1:** Demographic and laboratory characteristics in LN and Non-LN patients

	Non-LN (n=54)	LN (n=123)
Age (years)	37.13±13.43	36.91±13.27
Male, n (%)	4 (7.41%)	21 (17.1%)
WBC (×10^9^/L)	5.86±2.15	6.74±4.16
RBC (×10^12^/L)	3.87±0.54	3.99±0.72
HGB (g/L)	113.41±15.18	114.95±21.77
PLT (×10^9^/L)	189.91±100.19	205.08±95.66
BUN (mmol/L)	4.78±1.58	5.76±3.22*
Cr (umol/L)	54.83±11.09	62.36±19.53*
TP (g/L)	69.17±9.93	60.76±13.45*
Alb (g/L)	39.17±6.60	32.76±8.21*
IgG (g/L)	16.60±5.35	14.47±7.02
IgA (g/L)	3.12±1.23	3.04±1.20
IgM (g/L)	0.97±0.46	0.98±0.61
C_3_ (g/L)	0.73±0.31	0.66±0.31
C_4_ (g/L)	0.15±0.09	0.12±0.08*
Anti-ds-DNA antibody (IU/ML)	136.64±194.84	357.06±450.05*
SLEDAI	8.36±0.81	9.50±3.09

WBC: white blood cell; RBC: red blood cell; HGB: hemoglobin; PLT: platelet; BUN: Clinical Disease Activity Index; Cr: creatine; TP: total protein; Alb: albumin; Ig: immunoglobulin; C_3_: complement 3; C_4_: complement 4; SLEDAI: SLE disease activity index. Values are shown as mean ± *SD* unless otherwise noted. *p<0.05 compared to Non-LN group.
